# Corneal Endothelial Cell Loss after Ciliary Sulcus Placement of Ahmed Glaucoma Valve in Patients with Noninfectious Uveitic Glaucoma

**DOI:** 10.3390/jpm12122075

**Published:** 2022-12-16

**Authors:** Kaori Komatsu, Yosuke Harada, Tomona Hiyama, Ikuyo Sada, Kazuyuki Hirooka, Yoshiaki Kiuchi

**Affiliations:** Department of Ophthalmology and Visual Science, Graduate School of Biomedical Sciences, Hiroshima University, 1-2-3 Kasumi, Minami-ku, Hiroshima 734-8551, Japan

**Keywords:** uveitic glaucoma, Ahmed glaucoma valve, corneal endothelial cell density, intraocular pressure

## Abstract

This study was performed to investigate the corneal endothelial cell density (CECD) reduction and treatment efficacy in patients with uveitic glaucoma treated by the ciliary sulcus placement of the Ahmed glaucoma valve (AGV). This retrospective study included 27 eyes of 27 patients with noninfectious uveitis who underwent the sulcus placement of the AGV. Each patient underwent a clinical assessment including a CECD measurement before surgery and at 3, 6, 9, and 12 months after surgery. The mean CECD was 2431.4 ± 367.5 cells/mm^2^ at preoperative baseline and 2360.5 ± 391.3 cells/mm^2^ at 12 months (*p* = 0.074), with a reduction rate of 2.73 ± 9.29%. The CECD reduction was significantly greater in patients with unilateral uveitis than that with bilateral uveitis. The rate of successful intraocular pressure control was 88% at 12 months, and the number of intraocular pressure-lowering medications was significantly reduced (*p* < 0.001). The current study showed that the implantation of an Ahmed tube into the ciliary sulcus provided stable intraocular pressure control in patients with glaucoma secondary to noninfectious uveitis, and CECD reduction was moderate in most patients at 12 months.

## 1. Introduction

Uveitis is one of the leading causes of blindness, and uveitic glaucoma exacerbates its risk [1]. Elevated intraocular pressure (IOP) caused by uveitic glaucoma may contribute to this risk by the precipitation of inflammatory deposits such as inflammatory cells and fibrin in the trabecular meshwork and the effects of corticosteroids administered as part of the treatment [2]. There are also many other causes of elevated IOP, such as pupillary block caused by inflammation in the anterior chamber. The initial treatment of uveitic glaucoma involves the suppression of uveitis-induced inflammation by steroids and immunosuppressive drugs. If the condition remains progressive, however, the same treatment as for other types of glaucoma must be given, with the first step being IOP-lowering medications. When the effect of IOP-lowering medications on elevated IOP caused by uveitis is unstable even with multiple drug therapies, glaucoma surgery is performed [3]. The two typical surgical procedures are trabeculectomy with adjunctive antifibrosis and the implantation of glaucoma drainage devices.

For both trabeculectomy and the implantation of glaucoma drainage devices, many clinicians have great concern about corneal endothelial cell density (CECD) reduction. Moreover, ocular inflammation itself has been shown to adversely affect the corneal endothelium [4]. In particular, uveitic glaucoma was shown to be a risk factor for CECD reduction following trabeculectomy [5]. However, another study showed that uveitic glaucoma was not associated with CECD reduction after the sulcus placement of glaucoma drainage devices [6]. Information regarding CECD reduction after the implantation of glaucoma drainage devices in patients with uveitis remains limited, and a further accumulation of data is needed, especially in the Asian population.

In this study, we investigated the CECD reduction and treatment efficacy of the ciliary sulcus placement of the Ahmed glaucoma valve (AGV) in patients with uveitic glaucoma.

## 2. Patients and Methods

### 2.1. Patients

This retrospective study included patients with noninfectious uveitis who underwent the sulcus placement of the AGV at Hiroshima University Hospital from March 2017 to July 2021. Patients with preoperative CECD greater than 1500 cells/mm^2^ and those who were followed up for more than 12 months after the surgery were included. The exclusion criteria included contact lens use, ocular trauma, congenital abnormalities, and pre-existing corneal disease.

### 2.2. Objectives

The primary objective of this study was to evaluate endothelial cell loss after AGV placement as well as the efficacy of this treatment in patients with uveitic glaucoma. The secondary objectives were to assess the change in IOP, ocular inflammation, and IOP-lowering medications.

### 2.3. Data Collection

We collected the following data for patients treated with the AGV: age at uveitis onset and AGV implantation, duration of uveitis, CECD, type of uveitis (anterior versus intermediate, posterior, or panuveitis), involvement (unilateral or bilateral), diagnosis of uveitis, medical and surgical treatment history, and ophthalmological findings such as the best corrected visual acuity, IOP, anterior chamber cell grade, and laser flare photometry values (objective and quantitative measurements of aqueous humor protein levels in the anterior chamber). The classification and grading of uveitis were based on the 2005 Standardization of Uveitis Nomenclature criteria [7]. The CECD was measured by a specular microscope and autofocus device (Topcon SP-3000; Topcon Corporation, Tokyo, Japan). This automatic analysis can sometimes provide a misleading analysis of the cell margin, especially in patients with a lower CECD, leading to an inaccurate value; therefore, experienced technicians performed the analyses to confirm that the cell margins were properly recognized. The CECD was measured before surgery and at 3, 6, 9, and 12 months after surgery. In all examinations, the corneal endothelial cells were measured once at the center of the cornea. The IOP was measured with the iCare TA01i tonometer (Tiolat Oy, Helsinki, Finland). A relapse of uveitis was defined as a two-step increase in the level of inflammation, an increase from grade 3+ to 4+ in the anterior chamber or vitreous haze score, new cystoid macular edema or retinal/choroidal lesion activity, and/or a requirement for corticosteroid rescue therapy. The number of relapses per year was recorded before and after surgery using the following relapse rate scoring system: 0, no relapses; 1, one relapse; 2, two relapses; 3, three relapses; and 4, four or more relapses. An improvement in terms of ocular inflammation after AGV implantation was defined as a decrease in the number of relapses each year after the surgery. If the number of relapses after the surgery was the same as or worse than that before the surgery, the eye was considered to have no improvement.

### 2.4. Diagnosis

Sarcoidosis and Vogt–Koyanagi–Harada disease were diagnosed in accordance with international diagnostic criteria [8,9]. Sympathetic ophthalmia, a type of autoimmune bilateral granulomatous panuveitis that occurs after penetrating ocular trauma or surgery, was diagnosed clinically [10]. Multifocal choroiditis, a characteristic clinical manifestation of panuveitis that manifests as multiple pigmented chorioretinal lesions in the posterior and mid-peripheral retina without any associated systemic or infectious etiologies, was diagnosed in accordance with the diagnostic criteria of the Standardization of Uveitis Nomenclature working group [11]. Retinal vasculitis was diagnosed when a vascular alteration was observed by fluorescein angiography without any associated systemic, infectious, or ocular disorders or associated neoplasia [12]. Patients with suspected infection underwent polymerase chain reaction of the aqueous humor for herpes simplex virus, herpes zoster virus, and cytomegalovirus, and all cases (particularly all unilateral cases) were proven negative.

### 2.5. Surgery

A fornix-based conjunctival flap was made in the superotemporal or inferotemporal quadrant. The AGV (model FP7; New World Medical, Rancho Cucamonga, CA, USA) was gently inserted into the sub-Tenon space. The plate was attached to the sclera 10 mm posterior to the limbus. The tube was trimmed to an adequate length that allowed for visibility of the tube tips at the edge of the pupil. Viscoelastic material was injected through a side port between the iris and intraocular lens at the site of tube insertion. A 23-gauge needle was inserted between the iris and intraocular lens to create a scleral tunnel. The tube was then carefully inserted through the scleral tunnel without damaging the iris. After the tube tip was confirmed to be in the appropriate position without being plugged by the posterior iris, the tube was attached to the sclera with a 10.0 nylon suture. The scleral patch graft was sutured over the tube. The Tenon fascia and conjunctiva were closed with 8.0 Vicryl suture. The viscoelastic material was removed from the anterior chamber using a balanced salt solution (Alcon Laboratories, Fort Worth, TX, USA). All patients had achieved inflammatory quiescence at the time of surgery. Ocular inflammation during the perioperative period was controlled with topical/systemic corticosteroids or immunosuppressive drugs when necessary. All surgeries were performed by an experienced surgeon (Y.H).

### 2.6. Statistical Analyses

Statistical analyses were performed using JMP software version 15 (SAS Inc., Cary, NC, USA). If both eyes underwent AGV implantation, we chose the eye that underwent implantation first. Univariate comparisons between groups were conducted using the paired *t*-test and two-sided Student’s *t*-test for continuous variables. We performed a simple regression analysis to identify the risk factors for a postoperative decrease in the CECD. Stepwise multiple regression analysis was performed to determine factors associated with a decrease in the CECD. The secondary outcome measures in this study were IOP reduction and surgical failure. Surgical failure was defined as an IOP of >21 mmHg or IOP not reduced by 20% below the baseline value on two consecutive follow-up visits after 3 months, an IOP of <5 mmHg on two consecutive follow-up visits after 3 months, reoperation for glaucoma, or loss of light perception vision. A Kaplan–Meier survival curve was used for survival analyses to assess patients in whom surgery was successful (i.e., achieved successful IOP control). All data are reported as the mean ± standard deviation. A *p*-value of <0.05 was considered statistically significant.

## 3. Results

Table 1 shows the patients’ baseline characteristics. A total of 27 eyes of 27 patients with uveitic glaucoma underwent AGV implantation. The patients’ mean age was 67.4 ± 11.8 years, and they comprised 14 men and 13 women. The duration of uveitis before AGV insertion was 11.4 ± 13.9 years, and the follow-up period after surgery was 1.7 ± 0.5 years. The most common cause of uveitis was chronic iridocyclitis (10 eyes, 37.0%), followed by retinal vasculitis (5 eyes, 18.5%) and sarcoidosis (4 eyes, 14.8%). Twenty-seven eyes had a history of intraocular surgery. The most common type of surgery before AGV implantation was cataract surgery, in 24 eyes (88%), and glaucoma surgery with trabeculectomy, in 9 eyes (33%). Three eyes (11%) underwent concomitant cataract surgery with AGV implantation (Table 1). There were no inflammatory relapses in 24 of 27 eyes, whereas relapse occurred in 3 eyes following AGV implantation.

**Figure 1:**
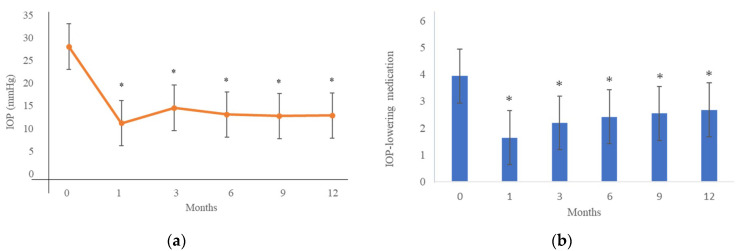
(**a**) Preoperative and postoperative IOP; (**b**) numbers of IOP-lowering medications. IOP, intraocular pressure. * *p* < 0.05 compared with baseline.

**Figure 2:**
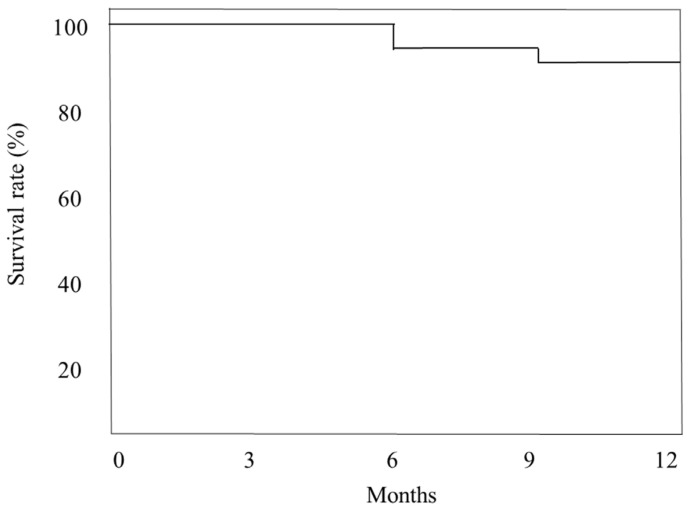
Kaplan–Meier survival curve of the surgical outcome.

The mean preoperative IOP was 28.7 ± 9.6 mmHg, and the IOP significantly decreased to 13.3 ± 4.5 mmHg at 12 months postoperatively (*p* < 0.001) (Figure 1a). The number of IOP-lowering medications decreased from 3.9 ± 0.7 preoperatively to 2.7 ± 1.1 at 12 months postoperatively (*p* < 0.001) (Figure 1b). Figure 2 shows a Kaplan–Meier survival curve of the surgical outcome; successful IOP control was defined as an IOP of 5 to 21 mmHg with or without IOP-lowering medications and a reduction of ≥20% from the preoperative level. At 6 months and at 12 months, IOP was successfully controlled in 92% and 88% of the patients, respectively.

Table 2 shows the reduction in CECD after surgery. The mean CECD was 2431.4 ± 367.5 cells/mm^2^ at preoperative baseline. The reduction rate at 6 and 12 months was 3.69 ± 13.67% and 2.73 ± 9.29%, respectively. The CECD was also measured in the contralateral eyes of patients with bilateral uveitis. Four patients who underwent AGV implantation in both eyes were excluded. Seventeen eyes were eligible for this analysis, two of which were not measurable because of bullous keratopathy. In the 15 remaining eyes, the mean decrease in the CECD at 12 months was 24.3 ± 168.2 cells/mm^2^ (reduction rate of 1.81 ± 8.39%).

To examine the factors associated with CECD reduction, the overall reduction rate was examined using multiple regression analysis. Only unilateral uveitis was associated with CECD reduction (*p* = 0.011) (Table 3). CECD reduction in patients with unilateral uveitis was almost three times greater than that in patients with bilateral uveitis (*p* = 0.010) (Table 4).

## 4. Discussion

The novelty of this study lies in the assessment of the treatment effect and CECD reduction after the ciliary sulcus implantation of the AGV in patients with secondary glaucoma limited to noninfectious uveitis with a follow-up of more than 12 months. By excluding eyes with infectious uveitis (such as anterior uveitis by herpes simplex virus, varicella zoster virus, or cytomegalovirus, all of which potentially affect the corneal endothelium), we evaluated how AGV implantation damages the corneal endothelium in patients with uveitis. We found that the surgical success of AGV implantation in the patients included in this study was 88% at 12 months postoperatively. The mean CECD was 2431.4 ± 367.5 cells/mm^2^ at preoperative baseline and 2360.5 ± 391.3 cells/mm^2^ at 12 months postoperatively (*p* = 0.074); the reduction rate was 2.73 ± 9.29%. Additionally, the risk of CECD reduction by AGV implantation was associated with unilateral uveitis.

The success rate in the present study was comparable to those previously reported when using the same definition of surgical success. According to the previous report, the success rate of IOP control with AGV implantation in patients with uveitis was 77% at 12 months when the definition of successful IOP control was an IOP of 5 to 21 mmHg with or without eye drops and a reduction of at least 25% of the preoperative IOP [13]. Lee et al. [14] compared the surgical outcomes between trabeculectomy with mitomycin C and AGV implantation with mitomycin C in eyes with uveitis and found similar postoperative success rates (81.8% vs. 81.2%, respectively) when surgical success was defined as an IOP decrease of more than 20%. Other studies showed higher surgical success in patients who underwent AGV rather than trabeculectomy [15,16]. Considering the possibility of postoperative hypotony, bleb revision or needling, which may be required later in trabeculectomy, AGV may have an economic benefit in the long term, despite the higher surgical cost of AGV compared with that of trabeculectomy.

The CECD in young adults is approximately 3000 cells/mm2 and decreases by a mean of 0.5 ± 0.6% per year with age [17]. The CECD was also reported to decrease secondary to ocular inflammation [4]. Among the patients with bilateral uveitis included in this study, the rate of corneal endothelial cell loss in the eyes of those who did not undergo AGV implantation was 1.81 ± 8.39% at 12 months. This rate was greater than that caused by aging, suggesting corneal endothelial cell loss due to uveitis.

The corneal endothelium is also damaged by intraocular surgery. Kim et al. [18] reported that in all types of glaucoma, the CECD reduction rate was 3.2% at 12 months after trabeculectomy compared with 12.3% after AGV insertion in the anterior chamber. Others reported that the rate of CECD loss was 2.5 to 8.6% at 12 months after AGV insertion in the ciliary sulcus [19,20,21]. These reports contained many types of glaucoma and less than 30% of the patients had uveitis glaucoma. Murakami et al. [6] reported a 7.3% reduction rate at 12 months after the placement of both the AGV and the Baerveldt glaucoma implant, and in patients with uveitic glaucoma, the reduction rate was 4.7% at 12 months. Therefore, uveitis has not been shown to be a factor associated with a reduction in the CECD after the placement of glaucoma drainage devices, and to the best of our knowledge, this is the first report of CECD reduction after the sulcus placement of AGV for uveitic glaucoma. Although the eyes in the present study were exposed to a risk of corneal endothelial damage caused by ocular inflammation or the AGV implantation procedure, the mean CECD reduction rate (2.73 ± 9.29% per year) did not exceed the previously reported reduction rates after AVG implantation. We think that the reasons for this were the ciliary sulcus insertion of the AGV in all cases and the control of ocular inflammation preoperatively and postoperatively in most cases (more than 88% of cases showed no deterioration of the relapse rate after AGV implantation). These data also support the notion that uveitis itself is not a risk factor for CECD reduction by AGV implantation as reported by Murakami et al. [6]. Although the number of patients and the follow-up period were limited, we think our data have considerable clinical significance.

However, this study revealed that unilateral uveitis was significantly associated with corneal endothelial damage by AGV implantation among the eyes with noninfectious uveitis. The reason for this remains unclear. One reason may be that an infectious etiology could not be completely ruled out in this study. In a previous study, the cytomegaloviral genome could not be detected by a single polymerase chain reaction in anterior uveitis with cytomegalovirus [22]. Thus, we could not completely rule out the possibility of infectious uveitis despite the fact that all cases of unilateral involvement were confirmed to have negative polymerase chain reaction results for herpes simplex virus, herpes zoster, and cytomegalovirus using aqueous humor and the ocular inflammation in these cases was improved by steroid therapy. The further accumulation of data is needed to clarify whether unilateral uveitis is a risk factor for CECD reduction after AGV implantation.

Our study had some important limitations. First, it was a retrospective study. Second, the number of patients was limited, and the follow-up period was short. The third limitation was the accuracy of the CECD measurement. A fluctuation in the CECD could not be considered because it was measured only once per visit. Finally, we could not completely exclude the possibility that cases of uveitis with an infectious etiology were mixed in the study population despite all cases being confirmed as negative for infectious uveitis by serological tests or the polymerase chain reaction of the aqueous humor.

## 5. Conclusions

The current study showed that the implantation of the AGV tube into the ciliary sulcus provided stable IOP control in eyes with glaucoma secondary to noninfectious uveitis, and the CECD reduction was moderate in most cases at 12 months. Importantly, however, a decrease in the CECD was more likely in patients with unilateral uveitis.

## Figures and Tables

**Table 1:** Patients’ baseline characteristics.

Patients (n)	27
Eye (n)	27
Age (y)	67.4 ± 11.8
Age at uveitis diagnosis (y)	56.0 ± 17.5
Follow up period until surgery (y)	11.4 ± 13.9
Gender (M/F)	14/13
Type of noninfectious uvea n. (%)	
Retinal vasculitis	5(18.5%)
Multifocal choroiditis	3 (11.1%)
Chronic iridocyclitis	10 (37.0%)
Sarcoidosis	4 (14.8%)
Vogt–Koyanagi–Harada disease	3 (11.1%)
Fuchs iridocyclitis	1 (3.7%)
Sympathetic ophthalmia	1 (3.7%)
Anterior uveitis n. (%)	11 (40.7%)
Bilateral/unilateral	21/6
Preoperative IOP (mmHg)	28.7 ± 9.6
Preoperative IOP-lowering medications (n)	3.9 ± 0.7
Preoperative intraocular surgeries (n)	
None	3
Cataract	24
Glaucoma surgery	9
Vitrectomy	7
Preoperative CECD (cells/mm^2^)	2431.4 ± 367.5
Preoperative central cornea thickness (μm)	494.0 ± 32.8
Preoperative number of relapses per year	0.9 ± 0.9
Preoperative flare measurement (ph/ms)	30.8 ± 11.8

Data are shown as n, n (%), or mean ± standard deviation. M, male; F, female; IOP, intraocular pressure; CECD, corneal endothelial cell density.

**Table 2:** Comparison of CECD in eyes treated by AGV implantation.

Follow-Up Period	N	CECD (cells/mm^2^)	Reduction (%) *	*p* Value ^a^
Baseline	27	2431.4 ± 367.5		
3 months	22	2307.7 ± 391.0	3.69 ± 13.67	0.119
6 months	26	2322.3 ± 378.7	4.28 ± 10.15	0.059
9 months	22	2306.4 ± 365.1	3.26 ± 10.93	0.103
12 months	27	2360.5 ± 391.3	2.73 ± 9.29	0.074

Data are shown as the mean ± standard deviation. * Percentage decrease in the CECD from baseline. ^a^ Compared with the baseline CECD (paired *t*-test). *p* values of <0.05 were considered statistically significant. AGV, Ahmed glaucoma valve; CECD, corneal endothelial cell density.

**Table 3:** Stepwise multiple regression analysis for factors associated with postoperative CECD changes.

	Univariate				Multivariate		
Factors	β	SE(β)	*p* Value		β	SE(β)	*p* Value
Age	−0.060	0.104	0.565				
Gender (Female)	0.411	1.221	0.738				
Type of uveitis							
Retinal vasculitis	−2.377	2.910	0.423				
Multifocal choroiditis	1.862	3.513	0.601				
Chronic iridocyclitis	−0.596	2.360	0.803				
Sarcoidosis	−2.030	3.150	0.526				
Vogt–Koyanagi–Harada disease	−1.729	3.513	0.627				
Anterior uveitis	−1.002	1.228	0.421				
Unilateral	3.896	1.247	0.004		−7.130	2.603	0.011
Age at uveitis diagnosis	0.055	0.069	0.431				
Follow up period until surgery	−0.129	0.085	0.143		−0.090	0.078	0.259
Preoperative intraocular surgery	−0.728	1.331	0.589				
Preoperative trabeculectomy	0.972	1.282	0.455				
Combined with cataract surgery	−0.223	1.445	0.878				
Preoperative laser flare	0.011	0.042	0.791				
Improvement of ocular inflammation	−1.357	1.260	0.291				
Preoperative IOP	0.076	0.128	0.557				
Preoperative CECD	0.001	0.003	0.803				
Preoperative IOP-lowering medications	1.107	1.751	0.532				
Anterior chamber depth	−4.801	3.450	0.177				

IOP, intraocular pressure; CECD, corneal endothelial cell density; β, regression coefficient; SE, standard error. *p* values of <0.05 were considered statistically significant.

**Table 4:** Comparison of CECD in patients with unilateral and bilateral uveitis.

Follow-Up Period	Unilateral (n = 6)		P ^a^	Bilateral (n = 25)		P ^b^	P ^c^
	CECD (cells/mm^2^)	Reduction (%)*		CECD (cells/mm^2^)	Reduction (%)*		
Baseline	2467.6 ± 363.7			2421.1 ± 522.5			
6 months	2084.4 ± 244.1	14.89 ± 9.62	0.021	2393.7 ± 387.1	1.10 ± 8.06	0.460	0.001
12 months	2198.5 ± 397.1	11.02 ± 7.19	0.012	2406.9 ± 386.7	3.70 ± 8.53	0.745	0.010

Data are shown as the mean ± standard deviation. * Percentage decrease in the CECD from baseline. ^a,b^ Compared with the baseline CECD (paired *t*-test). ^c^ Unilateral reduction rate vs. bilateral reduction rate (Student’s *t*-test). *p* values of <0.05 were considered statistically significant.

## Data Availability

All the relevant data used in this study were included in this manuscript.
